# A-to-I RNA editing enzyme ADAR2 regulates light-induced circadian phase-shift

**DOI:** 10.1038/s41598-018-33114-6

**Published:** 2018-10-04

**Authors:** Hideki Terajima, Hikari Yoshitane, Tomoko Yoshikawa, Yasufumi Shigeyoshi, Yoshitaka Fukada

**Affiliations:** 1 Department of Biological Sciences, The University of Tokyo School of Science, Hongo 7-3-1, Bunkyo-ku, Tokyo 113-0033 Japan; 2 Department of Anatomy and Neurobiology, Kindai University School of Medicine 377-2 Ohno-Higashi, Osakasayama City, Osaka 589-8511 Japan

## Abstract

In mammals, the central circadian clock is located in the suprachiasmatic nucleus (SCN) of the hypothalamus and it orchestrates peripheral clocks in the whole body to organize physiological and behavioral rhythms. Light-induced phase-shift of the SCN clock enables synchronization of the circadian clock system with 24-h environmental light/dark cycle. We previously found that *adenosine deaminase acting on RNA 2* (*Adar2*), an A-to-I RNA editing enzyme catalyzing rhythmic A-to-I RNA editing, governs a wide range of mRNA rhythms in the mouse liver and regulates the circadian behavior. In brain, ADAR2-mediated A-to-I RNA editing was reported to occur in various transcripts encoding ion channels and neurotransmitter receptors, which could influence neuronal function of the SCN. Here we show that ADAR2 plays a crucial role for light-induced phase-shift of the circadian clock. Intriguingly, exposure of *Adar2*-knockout mice to a light pulse at late night caused an aberrant phase-advance of the locomotor rhythms. By monitoring the bioluminescence rhythms of the mutant SCN slices, we found that a phase-advance induced by treatment with pituitary adenylyl cyclase-activating polypeptide (PACAP) was markedly attenuated. The present study suggests that A-to-I RNA editing in the SCN regulates a proper phase response to light in the mouse circadian system.

## Introduction

The circadian clock is a self-sustained time-measuring system that generates daily rhythms of behavior, physiology and gene expression with a period of approximately 24 h^1^. In mammals, a wide range of physiological and behavioral processes are orchestrated by the central clock in the SCN^2,3^. To maintain synchrony with the environmental conditions, the SCN receives photic information from the retina through retinohypothalamic tract (RHT) projection^4^, to which glutamate and PACAP predominantly contribute as neurotransmitters^5–7^. Photic stimulation received by the retina at early night induces phase-delay of the SCN clock, while the stimulation at late night induces phase-advance. These light-induced bidirectional phase-shifts of the circadian clock are thought to be evoked by acute induction of a subset of clock gene expression^3^, but the signaling pathway directing either phase-advance or phase-delay is still elusive.

The cell-autonomous circadian oscillation is driven by a mechanism based on transcriptional-translational feedback loops (TTFLs)^8–11^, where CLOCK and BMAL1 heterodimer activates transcription of *Per* and *Cry* genes^12–15^. Translated PER and CRY proteins bind to the CLOCK-BMAL1 complex and inhibit their transactivation^9^. In addition to the TTFLs, the importance of post-transcriptional regulation in the circadian clockwork has come under the spotlight in the last decade^16–20^. We recently demonstrated that *adenosine deaminase acting on RNA 2* (*Adar2*), an A-to-I RNA editing enzyme, generates circadian rhythmicities of a wide range of mRNAs and ADAR2 deficiency shortened the free-running period of the cellular and behavioral rhythms^21^. ADAR2 binds to double-stranded RNAs (dsRNA) and catalyzes hydrolytic conversion of adenosine (A) to inosine (I), a process termed A-to-I RNA editing^22,23^. When A-to-I RNA editing occurs in coding region of mRNA, it can cause an amino acid change of the coded protein because the translational machinery recognizes inosine as guanosine (G). Our previous study on the physiological role of ADAR2-mediated A-to-I RNA editing was mainly performed in the mouse liver^21^. On the other hand, it is known that A-to-I editing is most prevalent in the central nervous system and plays a significant role in neuronal function by modifying activities of receptors involved in neurotransmission^24,25^. In fact, dysregulation in A-to-I RNA editing causes neurological disorders^26^ such as amyotrophic lateral sclerosis (ALS)^27^ and glioblastoma multiforme^28^. These studies prompted us to investigate a role of ADAR2 for the circadian clock in the SCN.

Here we found that ADAR2 deficiency caused a larger phase-advance in response to light in the wheel-running activity rhythms, while leaving the phase-delay unaffected. The larger light-induced phase-advance in the *Adar2*-knockout mice was verified by jet lag experiments. Interestingly, PACAP signaling pathway was implicated in the mechanism of the ADAR2-regulated phase-advance in the SCN clock. Our data demonstrate an important role of ADAR2 in regulating the phase-advance of the circadian rhythms and suggest that RNA modification regulates the light input pathway in the SCN.

## Results

### ADAR2-mediated A-to-I RNA editing in the SCN

**Figure 1:**
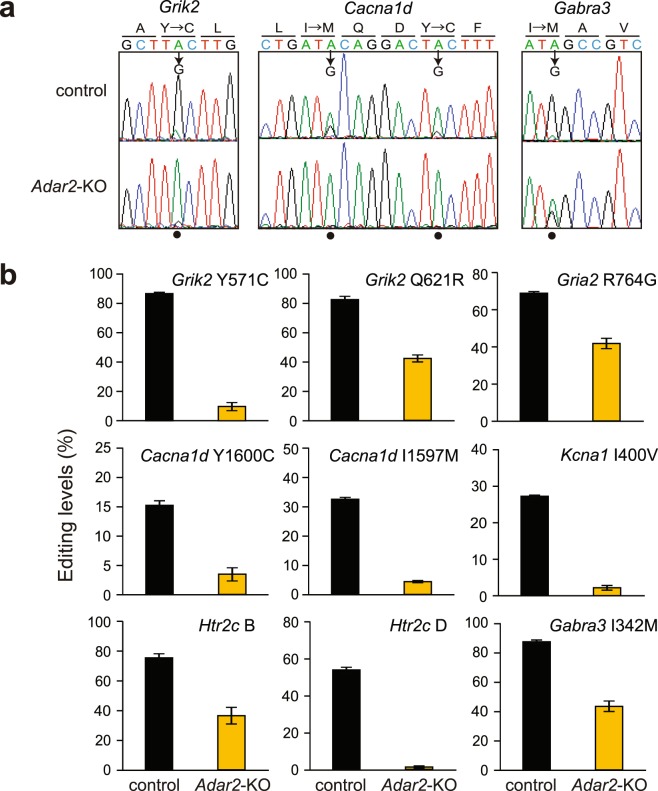
ADAR2-mediated A-to-I RNA editing in mouse SCN. (**a**) Direct sequencing chromatograms from RT-PCR products in control (top) and *Adar2*-KO (bottom) mouse SCN at CT22. The solid circles indicate the editing sites in the transcripts. (**b**) A-to-I RNA editing level at each editing sites in direct sequencing analysis (mean ± s.e.m.; *n* = 3).

ADAR-mediated A-to-I RNA editing is known to regulate activities of a range of ion channels and neurotransmitter receptors by recoding amino acid sequences (reviewed in refs^24–26^). Among three members of mammalian ADAR family, ADAR1, ADAR2 and ADAR3, the editing activity of ADAR3 has not been demonstrated yet. In the present study, contribution of ADAR2-mediated A-to-I RNA editing to the SCN neuronal function was investigated by using *Adar2*-deficient (*Adar2*^−/−^) mice. *Adar2*^−/−^ mice exhibit a postnatal lethal phenotype because Q607R substitution in GRIA2 does not occur due to a loss of the A-to-I editing activity^29^. The lethality is rescued by introducing a mutation replacing the codon for Q607 with arginine in *Gria2* (termed *Gria2*^*R/R*^)^29^. In this study, *Adar2*^−/−^*Gria2*^*R/R*^ and *Adar2*^+/+^*Gria2*^*R/R*^ mice were referred to as *Adar2*-knockout and control mice, respectively. We quantified A-to-I RNA editing levels [G/(A + G) (%)] in the SCN punch-out as the change of A peaks to G peaks in direct sequencing chromatograms of the RT-PCR products^30,31^. In control mice SCN, direct sequencing analysis detected substantial levels of A-to-I RNA editing in the transcripts coding ion channels and receptors, such as *glutamate receptor ionotropic AMPA2* (*Gria2*), *glutamate receptor ionotropic kainate 2* (*Grik2*), *calcium channel voltage-dependent L type alpha 1D subunit* (*Cacna1d*), *potassium voltage-gated channel shaker-related subfamily member 1 (Kcna1), 5-hydroxytryptamine receptor 2* *C* (*Htr2c*) and *gamma-aminobutyric acid A receptor subunit alpha 3* (*Gabra3*) (Fig. 1a, Supplementary Fig. 1a). Among these transcripts, ADAR2 deficiency significantly reduced the editing levels at particular sites (Fig. 1a,b, Supplementary Fig. 1a). Our results indicated that ADAR2 regulates considerable levels of the A-to-I editing of the mRNAs encoding these ion channels and receptors expressed in the SCN.

The SCN receives and transduces photic signals released from the terminal of RHT *via* several receptors and channels^3,32^. A major neurotransmitter, glutamate, released from the RHT terminals stimulates NMDA, AMPA (*Gria2*) and kainate (*Grik2*) receptors for membrane depolarization, which indirectly activates voltage-gated calcium channels such as Cav 1.3 (*Cacna1d*) expressed in the SCN neurons^3^. It should be noted that some of the A-to-I RNA editing events in these transcripts regulate their characteristics and neuronal activities^31,33–35^. These facts, together with our findings (Fig. 1), raise the possibility that ADAR2-mediated A-to-I RNA editing regulates the photic signal transduction in the SCN.

### Light-induced phase-shifts in *Adar2*-KO mouse

**Figure 2:**
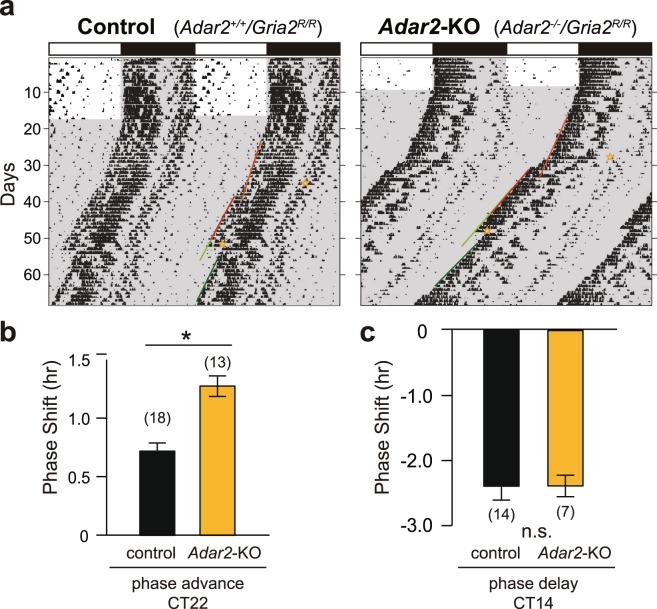
Effects of light pulses on the circadian phase at two different circadian times in *Adar2*-KO mice. (**a**) Representative double-plotted actograms of wheel-running activity. The area with gray shading indicates the dark period. The light pulses (30 min, 300 lux) indicated by yellow stars were given first at CT22 and subsequently given at CT14. The red and green lines indicate the onset of activity used to calculate phase-advance and phase-delay of locomotor activity rhythms, respectively. (**b**,**c**) Phase-shift induced by a light pulse given at CT22 (**b**) or CT14 (**c**) (mean ± s.e.m.; n.s. *P* ≥ 0.05, *P < 0.05 by Student’s *t*-test). The numbers of animals are indicated in parentheses.

*In vivo* role of ADAR2 in photic regulation of the SCN was examined by monitoring light-induced phase-shifts of the locomotor activity rhythms of *Adar2*-knockout mice. A 30-min light pulse given at early or late subjective night is respectively known to induce phase-delay or phase-advance of the circadian behavioral rhythms in mice^36^. Wheel-running activities of *Adar2*-knockout mice were recorded under the constant dark (DD) condition after entrainment under 12-h/12-h light/dark (LD) cycle. After two weeks in DD, mice were exposed to the 30-min light pulse at subjective night. When compared to control mice, *Adar2*-knockout mice showed significantly larger phase-advance by the light pulse given at CT22, where the activity onset was designated as CT12 (Fig. 2a,b). In contrast, no significant difference was observed in the phase-delaying effects induced by the light pulse at CT14 between control and *Adar2*-knockout mice (Fig. 2a,c). These observations revealed an important role of ADAR2 specifically in the light-induced phase-advance of the SCN clock. The normal phase-delay of the mutant mice by the light pulse given at CT14 suggests no significant difference in retinal light sensitivity between *Adar2*-knockout and control mice, though we cannot rule out the possibility that ADAR2 deficiency caused aberrance of photic signaling specific to the phase-advance.

**Figure 3:**
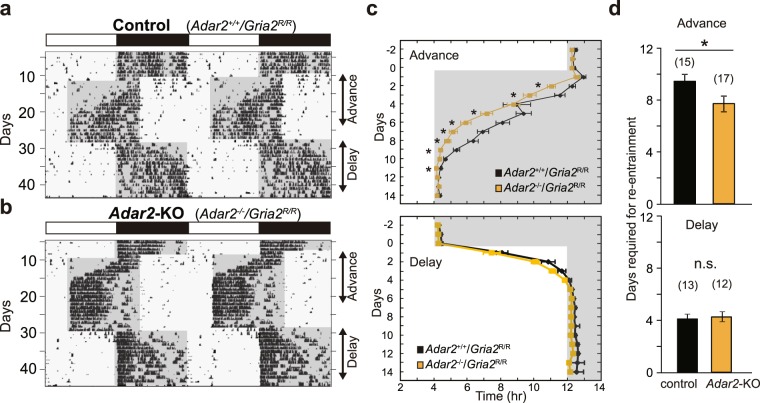
Re-entrainment of *Adar2*-KO mouse activities to a new LD cycle. (**a**,**b**) Representative double-plotted actograms of wheel-running activities in the jet lag paradigm. The area with gray shading indicates the dark period. (**c**) Activity onset in the jet lag paradigm with an 8-h advance and an 8-h delay (mean ± s.e.m.; *P < 0.05 by Student’s *t*-test). (**d**) Days required for re-entrainment to the new LD cycles after an abrupt shift of the 12-h/12-h LD cycles with an 8-h advance and an 8-h delay (mean ± s.e.m.; n.s. *P* ≥ 0.05, *P < 0.05 by Student’s *t*-test). The numbers of animals are indicated in parentheses.

The role of ADAR2 in the phase shifting mechanisms of the SCN was validated by jet lag experiments. Control and *Adar2*-knockout mice were subjected to an abrupt shift of the 12-h/12-h LD cycles with 8-h advance and subsequently to the reverse shift of 8-h delay (Fig. 3a,b). The re-entrainment to the new LD cycle was visualized by plotting the activity onsets of the locomotor rhythms (Fig. 3c). In control mice, the 8-h advance of the LD cycle evoked a gradual shift of the behavioral rhythms and it took 9.5 days in average for complete re-entrainment to the new LD cycle (Fig. 3a,c,d). Noticeably, it took 7.7 days in average in *Adar2*-knockout mice, thus showing faster re-entrainment to the advance of LD cycle (Fig. 3b–d). In contrast, ADAR2 deficiency caused no marked difference in the re-entrainment when the mice were subjected to an 8-h delay (Fig. 3a–d). These results are consistent with the light-pulse experiments showing that ADAR2 deficiency caused an increase of the phase-advance while having no measurable effect on the phase-delay (Fig. 2b,c).

Then we compared the A-to-I editing levels between CT14 and CT22 in transcripts encoding several receptors in the SCN punch-out, but no significant difference in the editing levels was found between the two time points (Supplementary Fig. 1b). In addition, the light pulse at CT22 caused no measurable change in the editing levels of these transcripts in both control and *Adar2*-knockout mice (Supplementary Fig. 1c). These results suggest that ADAR2 regulates photic signal transduction in a light-independent manner throughout the day. However, the possibility is not eliminated that we were unable to detect rhythmic (or light-dependent) change in A-to-I RNA editing levels because the SCN punch-out contains a mixed population of SCN neurons and many surrounding cells, and hence any changes of the RNA editing could be masked by the heterogeneity of their circadian phases.

Of note, we found no significant difference in not only the phase-delay but also the phase-advance between wild-type (*Gria2*^*Q/Q*^) and control (*Gria2*^*R/R*^) mice on the *Adar2*^+/+^ background (Supplementary Fig. 2a–c). These results indicate that the knock-in allele of *Gria2*^*R/R*^ has a marginal effect, if any, on the light-induced phase-shift.

### Effect of PACAP on the SCN slice rhythms of *Adar2*-KO mouse

**Figure 4:**
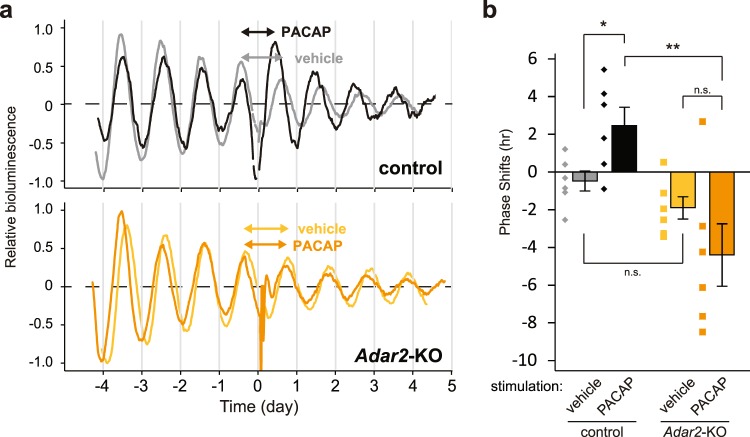
PACAP-induced phase-shift of bioluminescence rhythm recorded from the SCN slice in culture. (**a**) A representative set of bioluminescence rhythms of control (top) and *Adar2*-knockout (bottom) mouse SCN. The time of the PACAP application (1 μM, 1 h) was set to 0. The double-headed arrows indicate the peak intervals of the bioluminescence rhythms. (**b**) The phase-shifts induced by the PACAP application (mean ± SEM; *n* = 6; n.s. *P* ≥ 0.05, *P < 0.05, **P < 0.01 by Student’s *t*-test).

Light exposure of mice during the subjective night is known to stimulate retinal ganglion cells to release their neurotransmitters, glutamate and PACAP, from the RHT terminals^5–7^. These neurotransmitters induce the phase-shifts of the SCN rhythms for synchronization with external light/dark cycle^3^. To investigate the signaling pathway responsible for the ADAR2-regulated phase-advance, *Adar2*-knockout mice were crossed with PER2::LUC knock-in mice^37^, and the circadian oscillation of the bioluminescence signals were recorded from the SCN slices of PER2::Luc/Adar2^−/−^ mice. Compounds to be tested were dissolved in the recording medium and applied to the SCN slices for 60 min (60-min pulse treatment) at the late declining phase of the bioluminescence rhythms in order to mimic the photic stimulation at late night. When PACAP was applied to the SCN slice prepared from control mice, the next peak of the bioluminescence signal was significantly phase-advanced as compared to that after vehicle treatment with the recording medium (Fig. 4a, top, 4b), and this result is consistent with previous studies^7,38^. In contrast, ADAR2 deficiency abolished the phase-advancing effect of PACAP stimulation (Fig. 4a, bottom, 4b), indicating significant reduction of the PACAP-triggered signaling in the SCN of *Adar2*-knockout mice. Importance of the PACAP signaling in the phase shifting mechanism was demonstrated by previous studies describing that mutant mice deficient for PACAP^39,40^ or its specific receptor PAC1^41^ showed abnormal light-induced phase-shifts in the locomotor activities. Collectively, our data demonstrate that ADAR2 regulates the phase-shift mechanisms of the SCN clock through modifying PACAP signaling pathway. In addition, ADAR2 plays an important role in the appropriate light-induced phase-advance in mouse behavioral rhythms, though the detailed mechanism is yet to be determined.

## Discussion

Recent studies on the circadian oscillation ant its output mechanisms have shed light on the important roles of the post-transcriptional regulation, such as RNA methylation^18^, alternative splicing^19^, polyadenylation^42^ and miRNA-mediated gene silencing^20^. Our previous study demonstrated that A-to-I RNA editing enzyme ADAR2 is a key player for not only determining the circadian period of mouse behavioral rhythm but also generating a wide range of mRNA rhythms in the mouse liver^21^. Since A-to-I RNA editing events have been identified in key components of synaptic transmission and is known to have a strong impact on neuronal signaling^24^, we speculate that A-to-I RNA editing should play an important role in the SCN when receiving light signals from retina. The present study undertook to investigate the role of ADAR2 in light-dependent phase shifting mechanisms. We found augmentation of light-induced phase-advance in the wheel-running activity rhythms of *Adar2*-knockout mice (Fig. 2b). Our molecular level analysis indicates that PACAP signaling in the SCN should be regulated by ADAR2 (Fig. 4b).

It is reported that, in concert with glutamate, PACAP mediates transmission of light information to the SCN clock as a neurotransmitter released from the RHT terminal^43^. PACAP stimulates its specific receptor PAC1 to activate adenylyl cyclase signaling. Preceding studies on PACAP-deficient^39,40^ or PAC1-deficient^41^ mice demonstrated significant attenuation of phase-advance and phase-delay induced by light exposure. However, the A-to-I RNA editing databases^44,45^ in mouse transcriptome revealed no editing sites in PAC1, VPAC1 and VPAC2 receptors which are sensitive to PACAP. Here, it should be noted that binding of ADAR2 to dsRNAs by itself can modulate RNA processing independently of its RNA editing activity^46,47^. Such an editing-independent action of ADAR2 might contribute to expression levels of PACAP receptor(s) and/or the downstream signaling components, and consequently affect the light-induced phase-advance. In this study, we found that ADAR2 deficiency affected phase-advance but not phase-delay, suggesting that ADAR2 suppresses light signaling pathways specific to the phase-advancing process. It is also possible that the light signaling may be rhythmically regulated by ADAR2 in an editing activity-dependent or independent manner, because we found rhythmic expressions of *Adar2* and the related members in the SCN (Supplementary Fig. 1d).

As compared to control mice, *Adar2*-knockout mice exhibited a significantly larger phase-advance induced by a light pulse (Fig. 2b). In contrast, PACAP application did not significantly evoke the phase-shift in *Adar2*-knockout SCN slices, while PACAP caused the phase-advance in the SCN slices of control mice (Fig. 4). These apparently contradictory results could be associated with the cooperation of various receptors in ADAR2-regulated photic signaling pathways. Indeed, we found that several ion channels and receptors are subjected to A-to-I RNA editing in their mRNAs, which cause alteration in amino acid sequences (Fig. 1a,b, Supplementary Fig. 1a), and these changes might modulate neuronal activities in the SCN. For example, a recent study indicated that ADAR2-mediated RNA editing in voltage-dependent calcium channel (*Cacna1d*) modulates potential firing rates of the SCN slice^31^. In addition, A-to-I RNA editing at the R764G site in AMPA-type glutamate receptor (*Gria2*) regulates recovery rates from desensitization^34^, and editing at the Y571C and Q621R sites in kainate-type glutamate receptor (*Grik2*) changes calcium permeability of the receptors^35^. Interestingly, it is known that PACAP enhances the glutamatergic signaling by increasing glutamate-evoked currents^48^. Not only PACAP pathway but also these various ADAR2-regulated receptors and channels including glutamatergic pathways might orchestrate the phase-shifting mechanism in the SCN^49^. Further study is needed to explore how ADAR2 suppresses the light-induced phase-advance in mouse behavioral rhythm.

ADAR family members play key roles in adaptation to altered environmental conditions such as temperature, hypoxia and UV-irradiation in various species^50,51^. Collectively, our findings not only provide a new insight into the physiological role of ADAR2 in the SCN but also demonstrate that ADAR2 is important for synchronization between the circadian rhythms and environmental light/dark cycle through regulating the light-dependent phase shifting mechanism.

## Methods

### Animals

C57BL/6 J mice, PER2::LUC knock-in mice (C57BL/6 J background, obtained from The Jackson Laboratory)^37^ and *Adar2*^−/−^*/Gria2*^*R/R*^ mice (C57BL/6 J background, obtained from Mutant Mouse Regional Resource Centers (MMRRC))^29^, were housed in cages with commercial chow (CLEA Japan) and tap water available *ad libitum* under a controlled environment (temperature 23 ± 1 °C). All experiments were approved by the animal ethics committee of the University of Tokyo, and all experimental procedures were conducted in accordance with the University of Tokyo guidelines for use of experimental animals.

### Behavioral experiments

Young adult male mice were housed individually in cages equipped with running wheels and were entrained to the 12 h/12 h LD cycles for at least 2 weeks. The spontaneous locomotor activities were recorded by wheel-running revolutions in 5-min bins and the data were analyzed by using ClockLab software (Actimetrics). For light pulse experiments, the LD-entrained mice were released into the DD conditions for 3 weeks, and single light pulses (30 min, 300 lux) were given at circadian time (CT) 22, where the activity onset was designated as CT12. After the first light pulses, mice were maintained under the DD conditions for at least 15 days, and subsequently, second light pulses (30 min, 300 lux) were given at CT14. Phase-shifts were calculated from the phase-difference between the two regression lines, one fitted to 12 consecutive activity onsets immediately before the light pulse and the other to those after the light pulse excluding transient periods of first 3 days. For jet lag experiments, the entrained mice were subjected to an abrupt shift of the 12 h/12 h LD cycles with an 8-h advance and subsequently to the second shift of that with an 8-h delay.

### RNA preparation

Mouse SCN was isolated at each time point per day after 38 hr from the beginning of DD conditions (projected CT). Bilateral punches of the SCN were prepared from each frozen section (0.6 mm-thick) by using a stainless needle with an inside diameter of 0.6 mm. For quantitative RT-PCR and direct sequencing, total RNA was prepared from the SCN punch-out by using TRIzol (Thermo Fisher Scientic) according to the manufacturer’s protocol.

### Direct Sequencing

To quantify the A-to-I RNA editing levels, we performed direct sequencing analysis as described previously^21^. Briefly, total RNA was reverse transcribed by Go Script Reverse Transcriptase (Promega) with both anchored (dT)15 primers and random oligo primers. The cDNAs were amplified by KOD-Plus-Neo (TOYOBO) with gene specific primers (Supplementary Table S1, Fw1 and Rv1). Second PCR reactions were performed with the second sets of gene specific primers (Supplementary Table S1, Fw2 and Rv2) and with the first RT-PCR products (diluted 80-fold) as templates. Subsequently, the amplified cDNA fragments from RT-PCR were treated with ExoSAP-IT (USB) and directly sequenced with the gene specific primer, Fw2 or Rv2. The editing levels were quantified by measuring heights of A peaks (unedited) and G peaks (edited) and calculating percentage of the population edited at each site (100% × [G height/(A height + G height)]).

### Quantitative RT-PCR (qRT-PCR)

For quantification of gene expression, total RNA was reverse transcribed by Go Script Reverse Transcriptase (Promega) with both anchored (dT)15 primers and random oligo primers. The cDNA was subjected to real-time PCR with the gene specific primers (Supplementary Table S1).

### Real-time monitoring of luciferase expression in the SCN slices

Bioluminescence signals from the SCN cultures were recorded as described previously with minor modifications^52^. The coronal SCN slices (300 μm thick) were cultured on a membrane (Millicell-CM, Millipore) in a sealed 35-mm petri dish with the recording medium [DMEM (Sigma Aldrich) with B-27 Supplement, 0.1 mM luciferin (Promega), and 4.2 mM NaHCO_3_]. Bioluminescence signals were recorded at 37 °C in air with LumiCycle (Actimetrics). After recording the bioluminescence for 4 days, the SCN slices on a membrane were transferred to the medium with or without PACAP (final 1 μM) for an hour, and then washed twice with the recording medium for 5 min at 37 °C. After the wash, the SCN slices were transferred to the original culture medium. The PACAP stimulation was performed at a late declining phase of the luminescence rhythms. The raw data of bioluminescence rhythms were detrended by subtracting the 24-h moving average. To determine the degree of phase-shift induced by PACAP pulse treatment, the time points of three consecutive peaks of the bioluminescence rhythms before and after the PACAP application were fitted to regression lines. The two regression lines were extrapolated to a time when the pulse treatment was given, and the difference in time between the extrapolated time points were calculated as the phase difference.

## Electronic supplementary material

Supplementary information
