# Supplementary material for: A-to-I RNA editing enzyme ADAR2 regulates light-induced circadian phase-shift
Source: Sci Rep. 2018 Oct 4;8:14848. doi: 10.1038/s41598-018-33114-6 (PMC6172258; doi:10.1038/s41598-018-33114-6)
Supplement: Supplementary file 1 — Supplementary information [file 41598_2018_33114_MOESM1_ESM.pdf]

A-to-I RNA editing enzyme ADAR2 regulates light-induced circadian phase-shift

Hideki Terajima[1], Hikari Yoshitane[1], Tomoko Yoshikawa[2],
Yasufumi Shigeyoshi[2] and Yoshitaka Fukada[1,*]

[1]Department of Biological Sciences, School of Science,
The University of Tokyo, Hongo 7-3-1, Bunkyo-ku, Tokyo 113-0033, Japan.
[2] Department of Anatomy and Neurobiology, Kindai University Faculty of Medicine, Osaka-Sayama City, Osaka 589-8511, Japan.

[*]Correspondence should be addressed to:
Dr. Yoshitaka Fukada
Department of Biological Sciences,
School of Science, The University of Tokyo,
Hongo 7-3-1, Bunkyo-ku, Tokyo 113-0033,
Japan
Tel: +81-3-5841-4381
Fax: +81-3-5841-0763
E-mail: sfukada@mail.ecc.u-tokyo.ac.jp

Supplementary Material

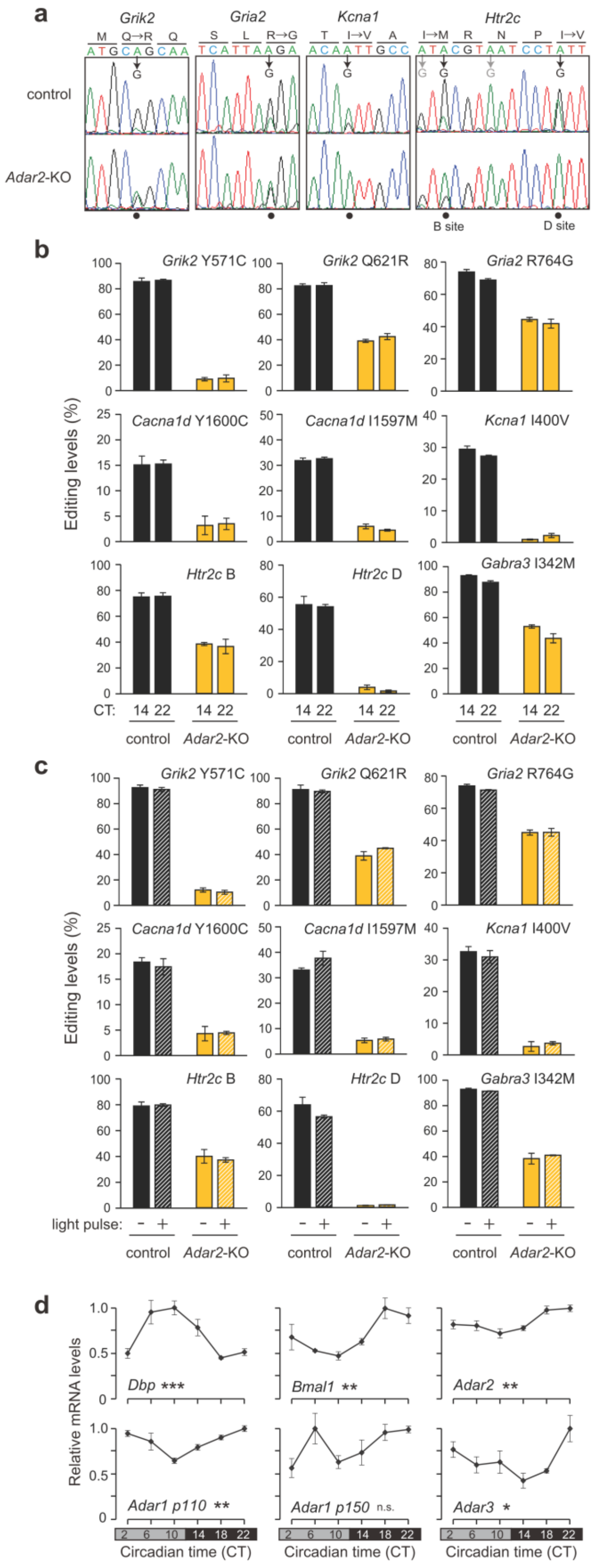

Supplementary Figure 1 ADAR2-mediated A-to-I RNA editing and temporal expression profiles of Adar family members in mouse SCN punch-out

(a) Direct sequencing chromatograms from RT-PCR products in control (top) and *Adar2*-KO (bottom) mouse SCN at CT22. The closed circles indicate the editing sites in the transcripts. (b) A-to-I RNA editing levels in direct sequencing analysis at CT14 and CT22 (mean ± s.e.m.; *n* = 3). The editing levels at CT22 were reproduced from Fig. 1b. (c) A-to-I RNA editing levels in direct sequencing analysis at CT23 with or without a light pulse (mean ± s.e.m.; *n* = 3). A 30-min light pulse was given at CT22 and RNA was extracted from the SCN 30 min after the light pulse. (d) Temporal expression profiles of *Dbp*, *Bmal1*, *Adar2*, *Adar p110*, *Adar p150* and *Adar3* in mouse SCN. mRNA rhythms of *Dbp* and *Bmal1* served as control for circadian rhythm. The signals were normalized to those of *Rps29* (mean ± s.e.m.; *n* = 3; * $P < 0.05$, ** $P < 0.01$, *** $P < 0.001$ and n.s. $P \geqq 0.05$ by one-way ANOVA).

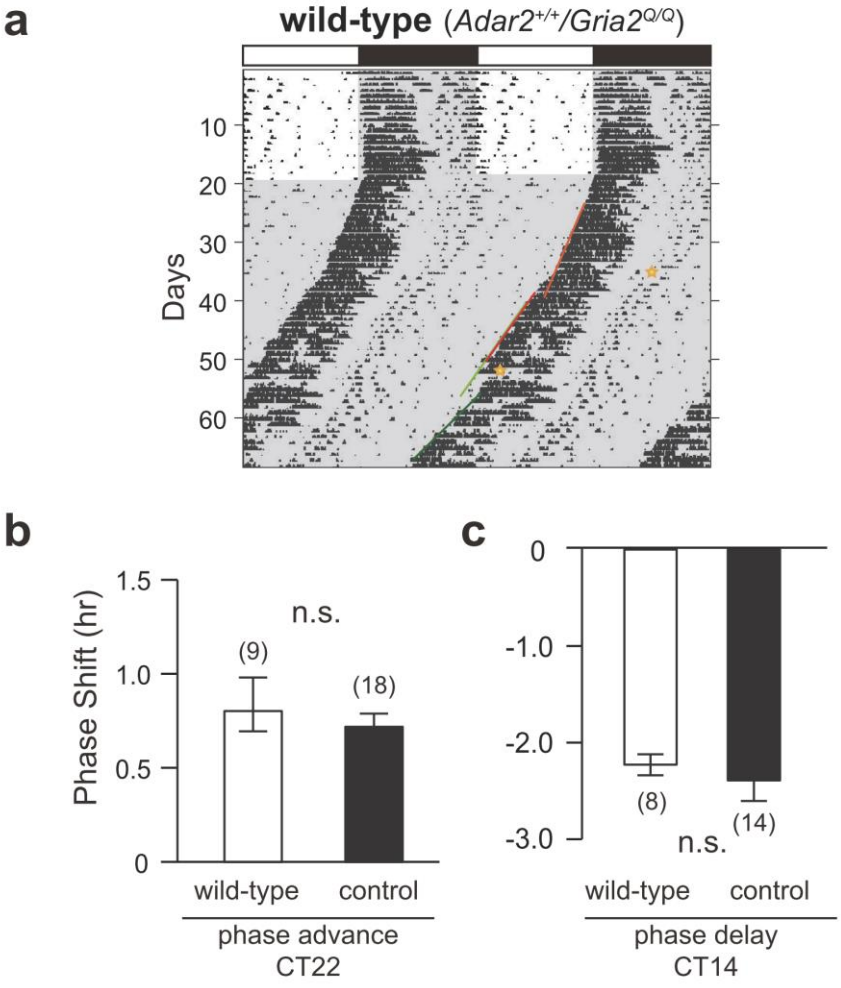

Supplementary Figure 2 Effects of a light pulse on the circadian phase at two different circadian times in wild-type mice

(a) Representative double-plotted actograms of wheel-running activity. The area with gray shading indicates the dark period. The light pulses (30 min, 300 lux) indicated by yellow stars were given at CT22 and subsequently given at CT14. The red and green lines indicate the onset of the wheel-running activity used to calculate phase-advance and phase-delay of the activity rhythms, respectively. (b, c) Phase-shifts induced by a light pulse given at CT22 (b) and CT14 (c) (mean ± s.e.m.; n.s. $P \geq 0.05$ by Student's *t*-test). The numbers of animals are indicated in parentheses.

Supplementary Table1: Gene specific primer sequences

primer name	sequence
Rps29-Fw	TGAAGGCAAGATGGGTCAC
Rps29-Rv	GCACATGTTCAGCCCGTATT
Adar2-Fw	TTGCCCTGAAGGAGTTTTG
Adar2-Rv	GAGGGCTTCTTGACTGGC
Adar p150-Fw	TCTCAAGGGTTCAGGGGAC
Adar p150-Rv	TACGACTGTGTCTGGTGAGGG
Adar p110-Fw	TTGGGACTAGCCGGGAAG
Adar p110-Rv	TACGACTGTGTCTGGTGAGGG
Adar3-Fw	AACACTGGCAGGAATCGTC
Adar3-Rv	TGATACACTTGGTCCCAGAGG
Dbp-Fw	AATGACCTTTGAACCTGATCCCGCT
Dbp-Rv	GCTCCAGTACTTCTCATCCTTCTGT
Bmal1-Fw	GCAGTGCCACTGACTACCAAGA
Bmal1-Rv	TCCTGGACATTGCATTGCAT
Grik2-Fw1	GCTCCACTGGCTATTACCTATG
Grik2-Rv1	AGCGGTATACGAAGAAATGATG
Grik2-Fw2	GTTCGTGAGAAGGTCATCG
Grik2-Rv2	GTGAAAAACCACCAAATGC
Gria2-Fw1	TGCAGTGTTTGATAAAATGTGG
Gria2-Rv1	GATGTAGAATACTCCAGCAACG
Gria2-Fw2	GACTTATATGAGGAGTGCAGAGC
Gria2-Rv2	TCAGACTGAGGGCACTGG
Cacna1d-Fw1	GAACCTGGAGCAAGCTAATG
Cacna1d-Rv1	TGGTTTGGAGTCTTCTGGC
Cacna1d-Fw2	CTGGAGCAAGCTAATGAAGAAC
Cacna1d-Rv2	TGGAGTCTTCTGGCTCGTC
Kcna1-Fw1	TATGAGGGAGTTAGGGCTGC
Kcna1-Rv1	TGATAGTAGAGGAGCTGCGG
Kcna1-Fw2	GGGCTGCTCATCTTTTTCC
Kcna1-Rv2	CTGAGGTCACTGTCAGAGGC
Htr2c-Fw1	GGCCAGCACTTTCAATAGTCGTG
Htr2c-Rv1	CAATCTTCATGATGGCCTTAGTCC
Htr2c-Fw2	ATGACAATAGGGGGCAAC
Htr2c-Rv2	TTCATGATGGCCTTAGTCC
Gabra3-Fw1	ATGACAACCCACTTTCATCTG
Gabra3-Rv1	CCTCTGGTACCTTCTTGCC
Gabra3-Fw2	TCATCTGAAGAGAAAAATTGGC
Gabra3-Rv2	CTGGTACCTTCTTGCCTTCC
